# Leptospirosis and its prevention: knowledge, attitude and practice of urban community in Selangor, Malaysia

**DOI:** 10.1186/s12889-019-6981-0

**Published:** 2019-05-22

**Authors:** Nurul Munirah Abdullah, Wan Mohd Zahiruddin Wan Mohammad, Mohd Nazri Shafei, Surianti Sukeri, Zawaha Idris, Wan Nor Arifin, Noramira Nozmi, Siti Nor Sakinah Saudi, Suhailah Samsudin, Abdul-Wahab Zainudin, Rukman Awang Hamat, Rosni Ibrahim, Siti Norbaya Masri, Suhainizam Muhammad Saliluddin, Aziah Daud, Malina Osman, Tengku Zetty Maztura Tengku Jamaluddin

**Affiliations:** 1 Department of Medical Microbiology and Parasitology, Faculty of Medicine and Health Sciences, Universiti Putra Malaysia, Selangor, Malaysia; 2 Department of Community Medicine, School of Medical Sciences, Universiti Sains Malaysia, 16150 Kubang Kerian, Kelantan Malaysia; 3 Health Promotion Unit, Penang State Health Department, Floor 7, Bangunan Persekutuan, Jalan Anson, 10400 Penang, Malaysia; 4 Unit of Biostatistics and Research Methodology, School of Medical Sciences, Universiti Sains Malaysia, 16150 Kubang Kerian, Kelantan Malaysia; 5 Health Department of Federal Territory Kuala Lumpur & Putrajaya, Jalan Cenderasari, 50590 Kuala Lumpur, Malaysia; 6 Department of Community Health, Faculty of Medicine and Health Sciences, Universiti Putra Malaysia, Selangor, Malaysia

**Keywords:** Leptospirosis, Knowledge, Attitude, Practice, Urban community

## Abstract

**Background:**

Leptospirosis is still endemic in Malaysia and has been prevalent in Selangor where cases have been underreported. Primarily, this was due to lack of awareness in the urban community in this region. This study determined the knowledge, attitude and preventive practice (KAP) of leptospirosis, and identified the significant predictors influencing KAP among urban community in Hulu Langat, Selangor.

**Methods:**

This cross-sectional study was conducted from 2015 to 2017 using validated questionnaire. Sampling methods included multistage cluster sampling, followed by simple random sampling to obtain 315 respondents. Descriptive analysis was performed to determine the KAP while χ^2^ and the subsequent logistic regression analysis were carried out to identify associations and predictors between variables.

**Results:**

Respondents were mainly Malaysian Bumiputra with a mean (Standard Deviation (SD)) age of 32.5 (13.0) years. Of 315 respondents, 80.3% (*n* = 253) had poor knowledge, 87.0% (*n* = 274) had good attitude, and 81.3% (*n* = 256) showed unacceptable practice towards leptospirosis and its prevention. Regression analysis identified age as the sole predictor influencing good knowledge (AOR 2.388; 95% CI = 1.298, 4.396; *p* = 0.005). Education level (AOR 2.197; 95% CI = 1.109, 4.352; *p* = 0.024) was also noted as the significant predictor influencing the overall practice.

**Conclusions:**

The urban community in Selangor showed a positive attitude in waste management despite having little knowledge regarding the disease itself. The study also discovered inadequacy in preventive practice, hence marking the importance of the proper integration of knowledge and attitude into forming an acceptable practice to reduce transmission of *Leptospira* among urban population in Malaysia.

## Background

Leptospirosis, a zoonotic disease caused by spirochetes such as *Leptospira interrogans*, occurs in a diverse epidemiological settings and affects underdeveloped, developing, as well as developed regions worldwide [1, 2]. Leptospirosis worldwide incidences were reported to be 0.1 to 1 per 100, 000 population and 10 to 100 per 100, 000 and could increase during seasonal outbreaks and among high-risk populations [3]. Incidence of human infection was higher in tropical areas of developing countries, and while it was generally endemic in humid tropics and subtropics climates, it was also possible to turn epidemic [4, 5].

Though not listed as a Southeast Asia (SEA) country predominantly affected by leptospirosis, it is still endemic in Malaysia after it became a notifiable disease in 2010 [6]. Nationwide, cases showed a steady increase for 10 years before reaching its peak at 2014 with 7806 cases and 92 deaths. In 2015, number of cases dropped to 5370 cases with only 30 deaths reported. Between the year 2004 until July 2015, the incidence rate in Malaysia was the highest in 2015 with 30.2 per 100, 000 population. During the same period, the mortality rate was the highest at 0.31 per 100, 000 population in 2014 nationwide [7]. In Selangor, 1030 and 1832 cases were reported in 2013 and 2014 respectively. Similar to the national report, number of cases in Selangor also dropped significantly to 879 cases in 2015. Cumulatively, there were two outbreaks with 14 cases occurred in Selangor over the course of 2015 where Hulu Langat district showed the highest number of cases [7].

Partly situated in the Klang Valley, Selangor is facing rapid urbanization and is densely populated. Average daily disposed solid waste collection in Selangor in 2013 was 4595 t, an increase of 743 t since 2008 [8]. Improper waste management operation became a common cause for the increase in animal carriers, especially rodents [9]. The proliferation of rodents and other carriers tend to contaminate fresh water and soil, leading to disease transmission at places highly frequented by public including recreational parks and heavily populated residential areas with close proximity to waste accumulation sites [10, 11].

Leptospirosis is a great depiction of the complexity surrounding the disease transmission between humans, animals and the ecosystem. Its prevention would require awareness from the public regarding its existence and general knowledge. This study focused on determining public’s awareness, including the ones who never contracted or ever heard of the disease in this region using knowledge, attitude and preventive practices (KAP) questionnaire.

## Methods

### Setting

This KAP study was conducted in Hulu Langat, Selangor. It is a quietly booming district located in the southeast of Selangor (2.9936° N, 101.7892° E), between Kuala Lumpur and Negeri Sembilan. It is the fifth largest district in Selangor State with an area of 840 km^2^ and a population of 1,141,880 [12]. It has equatorial climate, being hot and humid throughout the year. The district has both urban and rural settlements with majority of the population settling in towns near Kuala Lumpur.

### Study subjects

This study employed a cross-sectional study design, conducted from 2015 to 2017. Multistage cluster sampling method was done according to previous studies [13–15], where four sampling frames were outlined. Sampling element was then drawn from each frame to obtain the final list of residential streets in each selected residential area. The list of each residential street from 16 residential areas was created using a computer-based random number generator [16], excluding the industrial and business areas. Based on prevalence of good KAP within 5% of the true prevalence of 95% confidence and 10% non-response rate, the estimated sample size required for the study was 281. The sample size was calculated using Lemeshow and Fleiss formulae [17, 18].

All residents who lived in the selected street and fulfilled the criteria were invited to participate in the survey. These criteria include self-reported healthy individuals aged between 18 and 60 years old who have been residing in the neighborhood for at least 6 months. All eligible individuals who fit the inclusion criteria were approached to participate in the survey. If the individual was absent during the first house visit, second and third visits would be conducted again once during weekdays and weekends, if necessary. Individuals with known chronic illnesses and absent throughout the data collection period were excluded from the study. The questionnaire was self-administered and prior to data collection, respondents were briefed about the aim of the study and asked to provide a signed consent.

### Study instrument

This study made use of a set of validated questionnaire developed by a panel of experts working actively in leptospirosis consisting of epidemiologist, occupational health specialist, microbiologist, health educationist and medical statistician which developed a questionnaire consisting of six sections of close-ended questions concerning socio-demographic information, and KAP [19].

The leptospirosis-specific questions were developed through conducting focus groups representing the urban setting in Selangor to explore the insights of local community into leptospirosis and its prevention. An initial, qualitative investigation such as observation and focus group discussion would provide a more robust underpinning for the design of survey questions in exploring the large number of potential influences on behavioral and exposure risk [20]. This would have strengthened the questionnaire's validity and generated additional information. The sessions included questions regarding social background of participants, their knowledge on agent, mode of transmission, signs, symptoms, risk perception, and also preventive aspects of leptospirosis. The information gathered from the focus groups were used to develop constructs of the questionnaire. The average duration of answering the questionnaire was 30 mins.

This study was based on self-reported information so certain potential biases could be identified. Recall bias may be possible among the respondents while answering the questionnaire. Their responses towards certain sensitive matters such as personal hygiene and smoking habit could be the result of the apparent social desirability bias.

### Data analysis

The data were double-entered and analyzed using Statistical Package for Social Sciences (SPSS) version 22.0 (SPSS Inc., Chicago, IL, USA). Proportions of level of KAP of respondents on leptospirosis were calculated and presented as frequencies (%). χ^2^ test was used to analyze the association between two categorical variables (KAP and socio-demographic characteristics). *P*-value below 0.05 was considered statistically significant at 95% confidence interval (CI). Then, significant associations were further tested with logistic regression analysis to determine the adjusted odd ratios (AOR) and 95% CI of predictors influencing the outcome variables (good knowledge, good attitude, and good practice) among respondents.

## Results

### Socio-demographic characteristics

The response rate was 92.6% where out of the total 340 respondents identified, 315 individuals agreed to participate in the survey. Distribution of ethnicity was led by Malay respondents (81.0%) followed by other ethnicities with the mean (Standard Deviation (SD)) age of 32.5 (13.0) years old. There were slightly higher male respondents (51.5%) than female respondents. Almost 67 % of respondents went through tertiary education [21] and mean (SD) income was MYR 1951.75 (4536.94). The income group was further classified into ‘low income’ (less than MYR 1950) and ‘high income’ (MYR 1950 and above) [22].

### Knowledge on leptospirosis

Mean (SD) percentage score for knowledge was 59.42 (24.75). Although most respondents claimed that they have heard about the rat urine disease (85.4%), about 80 % (*n* = 253) of the 315 respondents had poor overall knowledge on leptospirosis. According to sections, majority of them answered correctly for general knowledge section while the rest of them did not know the answers. Seventy-one percent (*n* = 225) of respondents thought modes of transmission of leptospirosis were through contaminated food and drinks. For signs of leptospirosis, almost 83 % (*n* = 261) of them chose fever over myalgia and jaundice. For leptospirosis complications, 82 % (*n* = 257) of respondents chose death over breathing difficulty, kidney failure and liver damage. For prevention section, majority of them (87.9%) would avoid swimming in contaminated water bodies while only 61 % (*n* = 193) would wear gloves while working (Table 1).

**Table 1:** Distribution of knowledge items on leptospirosis

	*n* = 315
Knowledge items	Correct n (%)^a^
General knowledge:	
1. Rat urine disease is also known as leptospirosis	104 (33.0)
2. This disease is caused by bacteria	212 (67.3)
3. Leptospirosis is a zoonotic disease	202 (64.1)
4. Can be detected through blood test	177 (56.2)
5. Modes of transmission:	
i) Body wound	175 (55.6)
ii) Eyes	100 (31.7)
iii) Nose	120 (30.0)
iv) Mouth	138 (43.8)
v) Contaminated food	225 (71.4)
vi) Contaminated drink	225 (71.4)
vii) Handshake with infected person	131 (41.6)
6. Signs:	
i) Fever	261 (82.9)
ii) Myalgia	203 (64.4)
iii) Jaundice	122 (38.7)
7. Complication:	
i) Death	257 (81.6)
ii) Breathing difficulty	129 (41.0)
iii) Kidney failure	121 (38.4)
iv) Liver damage	115 (36.5)
8. Prevention:	
i) Make sure household compound free of waste	248 (78.7)
ii) Avoid wading in flooding area	213 (67.6)
iii) Keep personal hygiene	242 (76.8)
iv) Drink clean water	246 (78.1)
v) Wear gloves while working	193 (61.3)
vi) Avoid swimming in contaminated water body	277 (87.9)

^a^Percentage of respondents who gave the correct answers

### Attitudes towards leptospirosis

Attitude items consisted of 13 statements depicting good and unacceptable attitude towards leptospirosis prevention. The mean (SD) percentage score for attitude was 85.32 (8.82). Descriptive analysis showed 87 % (*n* = 274) of them had good attitude towards leptospirosis and its prevention. According to each attitude item, majority of respondents had good attitudes. However, there were 45.7% of them who figured that they would not be worried to wade in the flood water (Table 2).

**Table 2:** Distribution of attitude items on leptospirosis

	*n* = 315
Attitude items	Good attitude n (%)^a^
I will wear gloves when I handle waste	224 (71.1)
I will make sure waste bin is always covered	287 (91.1)
I have to cooperate with the health authority to prevent and control leptospirosis	277 (87.9)
I will make sure my family is involved to clean the house	276 (87.6)
I need to inform to the health authority if there is any leptospirosis case	284 (90.2)
I will make sure my family does not swim at contaminated water bodies	253 (80.3)
I am not worried to wade in the flood	144 (45.7)
I am oblivious of any presence of rodents around my house	263 (83.5)
I will inform to the health authority if I suspect any leptospirosis cases	249 (79.0)
I do not mind if my house is dirty	260 (82.5)
I need to see a doctor if I am having a fever during leptospirosis outbreak	270 (85.7)
I need to wear personal protective equipment (PPE) when handling waste	251 (79.7)
I am not worried if I am not wearing PPE such as boots, face mask etc. when handling waste	200 (63.5)

^a^Percentage of good attitude of respondents whom answered “strongly agree” or “agree” for attitude that they should have agreed and “strongly disagree” or “disagree” for attitude that they should not have agreed

### Preventive practice towards leptospirosis

Practice items consist of 17 statements depicting good and unacceptable preventive practice among respondents. The mean (SD) percentage score for practice was 64.31 (12.19). In overall, majority of respondents had unacceptable preventive practice (81.3%). Nevertheless, good practice was observed in several items including avoiding to eat or drink while handling waste (93.0%), choosing a clean restaurant (92.4%), and keeping foods in a covered container (91.1%). However, only 19 % (*n* = 60) of respondents would wear gloves and about 14 % (*n* = 46) would wear latex boots while handling waste (Table 3).

**Table 3:** Preventive practices on leptospirosis among respondents

		*n* = 315
Preventive Practices^*^	Good n (%)	Unacceptable n (%)
I make sure there is no rodent within the perimeter of my house	255 (81.0)	60 (19.0)
I went for a picnic at a waterfall / river / lake that was confirmed with leptospirosis (within the last 6 months)	234 (74.3)	81 (25.7)
I made sure my house is free from any waste	257 (81.6)	58 (18.4)
I get rid of the waste even when I have wounds/cuts on my hands/legs	197 (62.5)	118 (37.5)
I eat/ drink while handling the waste	293 (93.0)	22 (7.0)
I wash my hands with soap after handling the waste	268 (85.1)	47 (14.9)
I wear the following PPE while handling the waste:		
i) gloves	60 (19.0)	255 (81.0)
ii) latex boots	46 (14.6)	269 (85.4)
iii) long-sleeve shirt	144 (45.7)	171 (54.3)
I keep my food in a covered container	287 (91.1)	28 (8.9)
I consult a doctor when I have a fever during leptospirosis outbreak	194 (61.6)	121 (38.4)
I cover the waste bin to prevent rodent	281 (89.2)	34 (10.8)
I wash the can before I drink	210 (66.7)	105 (33.3)
I wash the cooking tools before I use them	280 (88.9)	35 (11.1)
I choose a clean restaurant	291 (92.4)	24 (7.6)
I put on plaster on wounds/small cuts when I am handling waste	222 (70.5)	93 (29.5)
I wade in floods without using PPE	162 (51.4)	153 (48.6)
I put on a plaster on small cut when I wade through flood water	61 (19.4)	254 (80.6)
I smoke while handling the waste	167 (53.0)	148 (47.0)

^*^The mean (SD) preventive practice score was 64.31 (12.19)

### Relationship between respondents’ socio-demographic characteristics with KAP towards leptospirosis

Chi square analysis showed that there were significant associations between ‘age’ with ‘knowledge’ and ‘education level’ with ‘preventive practice’. Subsequently, logistic regression was performed to predict the effect of ‘age’ and ‘education level’ on the likelihood that respondents have for having good knowledge and good preventive practice respectively. The two variables were included in the final model testing for being the only significant associations by the previous Chi square analysis. Respondents below 32 years were more likely to have good knowledge than those who were 32 years and above (AOR 2.388; 95% CI = 1.298, 4.396; *p* = 0.005). As for education level and preventive practice, logistic regression showed that those with higher education would have better preventive practice than those who were without higher education (AOR 2.197; 95% CI = 1.109, 4.352; *p* = 0.024) (Tables 4, 5).

**Table 4:** Association between socio-demographic characteristics with knowledge on leptospirosis

Variable	Knowledge Level:		*p*-value	Prevalence Ratio	95% CI	
	Poor (%)	Good (%)			Lower	Upper
Age**						
< 32 years old	133 (74.7)	45 (25.3)	0.004^a*^	2.037	1.221	3.398
≥ 32 years old	120 (87.6)	17 (12.4)				
Gender						
Male	128 (79.5)	33 (20.5)	0.710^a^	1.088	0.696	1.702
Female	125 (81.2)	29 (18.8)				
Ethnicity						
Bumiputra	207 (78.4)	57 (21.6)	0.053^a^	2.202	0.929	5.223
Non-Bumiputra	46 (90.2)	5 (9.8)				
Education Level						
Low education	164 (77.7)	47 (22.3)	0.099^a^	0.648	0.380	1.102
High education	89 (85.6)	15 (14.4)				
Monthly income (MYR)^†^						
Low income	115 (79.9)	29 (20.1)	0.852^a^	1.044	0.667	1.632
High income	138 (80.7)	33 (19.3)				

^a^ Chi square test; *Significant by chi-square test at *p* < 0.05; **Significant by logistic regression for age (AOR 2.388; 95% CI = 1.298, 4.396; *p* = 0.005); ^†^ Malaysian Ringgit

**Table 5:** Association between socio-demographic characteristics with preventive practice towards leptospirosis

Variable	Practice level		*p*-value	Prevalence Ratio	95% CI	
	Unacceptable (%)	Good (%)			Lower	Upper
Age						
< 32 years old	146 (82.1)	32 (17.9)	0.650^a^	0.897	0.563	1.432
≥ 32 years old	110 (80.0)	27 (20.0)				
Gender						
Male	125 (77.6)	36 (22.4)	0.091^a^	1.497	0.932	1.015
Female	131 (85.1)	23 (14.9)				
Ethnicity						
Bumiputra	213 (80.7)	51 (19.3)	0.543^a^	1.232	0.622	2.437
Non-Bumiputra	43 (84.3)	8 (15.7)				
Education Level**						
Low education	164 (77.7)	47 (22.3)	0.022^a*^	1.930	1.071	3.478
High education	92 (88.5)	12 (11.5)				
Monthly income (MYR) ^†^						
Low income	121 (84.0)	23 (16.0)	0.250^a^	0.759	0.472	1.219
High income	135 (78.9)	36 (21.1)				

^a^ Chi square; *Significant by chi-square at *p* < 0.05; **Significant by logistic regression for education level (AOR 2.197; 95% CI = 1.109, 4.352; *p* = 0.024); ^†^ Malaysian Ringgit

## Discussion

Various previous studies have utilized the KAP dimensions to determine predictors within the high risk groups such as town service workers and food handlers [23–26]. Nevertheless, studies exploring the KAP among non-high risk groups, especially in urban areas has never been conducted in this country prior to the commencement of this study. Then, following the findings of the current study and another KAP study in rural Selangor [33], leptospirosis intervention program was finally conducted and its effectiveness in improving KAP among wet market workers in Kelantan was proven. KAP scores became significantly higher in the intervention group than those of control group [27].

 The study aimed to determine the effectiveness of Leptospirosis Health Intervention Program (LHIP) in improving knowledge, attitude, belief and practice towards leptospirosis among wet market workers in Kelantan [27]. It was conducted in two main wet markets in Kelantan involving 116 participants in each control and intervention groups. The health education intervention was based on Leptospirosis Health Intervention Module. The knowledge, attitude, belief and practice scores were measured before and 6 weeks after the intervention to examine the effect of the program. Despite showing being familiar with the word “rat urine disease” or leptospirosis, majority of respondents in this study had limited specific knowledge on leptospirosis. Interestingly, this finding was similar to several other studies conducted both in urban and rural settings. For urban setting, studies from Trinidad and Tobago [28], Puerto Rico [29], Jamaica [30], Argentina [31], India [32] and Malaysia [25, 26] showed similar results. Meanwhile, a recent study in rural setting in Malaysia discovered most respondents had poor knowledge level as well [33]. Another recent study in India that combined both rural and urban respondents also discovered similar finding on the limited knowledge regarding leptospirosis, or the lack thereof [39]. This state of knowledge of urban and rural community on leptospirosis suggested an urgent need for repeated health education, especially for those with low education [34].

A large proportion of respondents had shown a good attitude. Positive attitude in waste management was observed among respondents in making sure the waste bin always covered, wearing gloves when handling waste, and wearing PPE when handling waste. Similar attitude was found among the local town service workers where positive attitude was observed in 91 % of the study participants regarding waste management. It was also reported that 87 % of respondents were concerned if the waste bin in their house was not covered [25, 26]. On the contrary, the study conducted in rural area  of Selangor revealed that 90 % of respondents showed negative attitude towards leptospirosis prevention [33]. Likewise, the study in Brazil identified that only less than half of respondents thought it was necessary to close the sewers and improve trash collection service to avoid waste accumulation [11].

Then, majority of respondents generally had unacceptable practice in leptospirosis prevention. Nevertheless, good practice could still be observed in several items where a narrow majority of respondents had good practice on avoiding to eat or drink while handling waste. Nevertheless, unsatisfactory practice was also observed among municipal workers in India [35] and town service workers in Malaysia [25]. The study in rural area of Selangor also showed majority of respondents had unsatisfactory preventive practice in overall [33]. The fact that respondents showed good practice in certain aspects could be due to the similarity of general preventive measures among certain infectious diseases, for instance typhoid and dengue. However, the importance of certain practices needs to be emphasized more than others. Public awareness towards the risk of unprotected wounds to bacterial infections while handling domestic waste needs to be raised, especially in terms of putting on PPE to provide adequate protection against *Leptospira*. Since putting on the full set of PPE could be burdensome in regular households, they should have at least worn one equipment that would provide adequate protection at the household level such as rubber gloves.

According to regression analysis, age was identified as the significant predictor influencing good knowledge of respondents towards leptospirosis and its prevention. This was in contrast to the KAP study conducted in the rural setting where ethnicity was the only significant predictor for influencing their knowledge level [33]. In this study, respondents below 32 years old were three times more likely to display good attitude and almost three times more likely to have good knowledge on leptospirosis. However, in order to initiate health-seeking behavior, having good knowledge per se is not adequate [36]. A proper set of attitudes must follow suit, and individual’s perceived benefit must override their perceived barrier in order to promote behavior change [37]. Being more technological savvy, younger age group also tend to rely on internet sources in gaining easy access to information about everything, including infectious disease. Nevertheless, not all information or knowledge accessed from the internet are necessarily correct and reliable. Thus, having endless source of information does not guarantee the accuracy of such knowledge.

Education level became the sole predictor to influence the good practice of respondents in this study. Similarly, the study in Philippines discovered high education level was associated with higher preventive practice among agricultural workers as compared to non-agricultural workers [38]. Meanwhile, in a recent study in Madurai district of India, education level proved to have significant impact on knowledge and attitude of urban residents, but their practice still did not improve with education [39]. Nonetheless, literatures discussing the relationship between education levels with specific preventive practice are still limited. In fact, various studies found no significant association between education status and preventive practice in Malaysia and other countries [25, 33, 35]. Instead of education level, these studies found association between type of occupation and preventive practice. Agricultural workers was shown to have significantly poor practice than non-agricultural workers while job category was associated with preventive practice of the respondents [35, 38]. The prediction supported the general idea that education level plays a crucial role in manifestation of good practice among the public.

Some of the limitations of this study include further exploration of reasons behind the involvement and non-involvement of the public in a particular prevention practice. Further investigation of this could be done qualitatively through open-ended questionnaires that would give respondents a freedom to express their opinions in detail. Additionally, social desirability bias could occur, for instance while determining the attitudes of the respondents regarding leptospirosis prevention. Respondents might give a response that they thought would be more acceptable to the interviewer rather than revealing their actual opinion. This could have been overcome by using self-administered or audio-assisted interview questionnaires for data collection.

## Conclusions

This study identified inconsistencies in KAP of respondents. Their knowledge and preventive practice were not adequate to be considered as good. However, positive attitude was observed in the waste management aspect of leptospirosis prevention. This indicates the importance of proper integration of knowledge and attitude in translating into an acceptable practice to reduce leptospirosis transmission. The general public who has little to no risk of getting leptospirosis in the normal condition will benefit the most from this study. Based on the findings, the right intervention can be tailored to the right population to instil the awareness and importantly the right knowledge regarding leptospirosis and its prevention. This will reduce the chances of the low risk individuals from becoming high risk individuals through elimination or reduction of risk factors by health intervention.

## Abbreviations

AOR: Adjusted Odd Ratio
CI: Confidence Interval
KAP: Knowledge, attitude and practice
MYR: Malaysian Ringgit
PPE: Personal Protective Equipment
SD: Standard Deviation
SEA: Southeast Asia
SPSS: Statistical Package for Social Sciences
